# Adherence, helpfulness and barriers to treatment in juvenile idiopathic arthritis – data from a German Inception cohort

**DOI:** 10.1186/s12969-023-00811-0

**Published:** 2023-04-12

**Authors:** Sabine Kirchner, Jens Klotsche, Ina Liedmann, Martina Niewerth, Debbie Feldman, Frank Dressler, Ivan Foeldvari, Dirk Foell, Johannes-Peter Haas, Gerd Horneff, Anton Hospach, Tilmann Kallinich, J. B. Kuemmerle-Deschner, Kirsten Moenkemoeller, Frank Weller-Heinemann, Daniel Windschall, Kirsten Minden, Claudia Sengler

**Affiliations:** 1 Deutsches Rheuma-Forschungszentrum Berlin, Leibniz Institute, Epidemiology Unit, Pediatric Rheumatology, Charitéplatz 1, 10117 Berlin, Germany; 2 Faculty of Medicine, School of Rehabilitation, Université de Montréal, Montreal, QC Canada; 3 Clinic for Paediatric PneumologyAllergology and Neonatology, Children’s Hospital, Medical School Hannover, Hannover, Germany; 4 Hamburg Centre for Pediatric and Adolescence Rheumatology, Paediatric Rheumatology, Hamburg, Germany; 5 Department of Paediatric Rheumatology and Immunology, University Hospital Muenster, Muenster, Germany; 6 German Centre for Child and Adolescent Rheumatology, Paediatric Rheumatology, Garmisch-Partenkirchen, Germany; 7 Asklepios Klinik St. Augustin, St. Augustin, Germany; 8 Department of Paediatric and Adolescent Medicine, University Hospital of Cologne, Cologne, Germany; 9 Olga Hospital, Department of Pediatrics, Stuttgart, Germany; 10 Department of Pediatric Respiratory Medicine, Immunology and Critical Care Medicine, Charité – Universitätsmedizin Berlin, Corporate Member of Freie Universität Berlin and Humboldt- Universität Zu Berlin, Berlin, Germany; 11 Division of Pediatric Rheumatology and Autoinflammation Reference Center Tübingen, Department of Pediatrics, University Hospital Tübingen, Tübingen, Germany; 12 Kliniken Köln - Kinderkrankenhaus Amsterdamer Str, Paediatric Rheumatology, Cologne, Germany; 13 Klinikum Bremen-Mitte, Prof-Hess-KinderklinikPaediatric Rheumatology, Bremen, Germany; 14 Clinic for Paediatric and Adolescent Rheumatology, Northwest German Center for Rheumatology, St. Josef Stift Sendenhorst, Sendenhorst, Germany; 15 University of Halle-Wittenberg, Halle, Germany

**Keywords:** Adherence, Medication, Exercise, Treatment, Juvenile idiopathic arthritis

## Abstract

**Objectives:**

To develop and evaluate German versions of the Parent Adherence Report Questionnaire (PARQ) and Child Adherence Report Questionnaire (CARQ) and to evaluate adherence in patients with juvenile idiopathic arthritis (JIA).

**Methods:**

The PARQ and CARQ were translated into German, cross-culturally adapted and administered to patients (age ≥ 8 years) and their parents enrolled in the Inception Cohort Study of newly diagnosed JIA patients (ICON).

The psychometric issues were explored by analyzing their test–retest reliability and construct validity.

**Results:**

Four hundred eighty-one parents and their children with JIA (*n* = 465) completed the PARQ and CARQ at the 4-year follow-up. Mean age and disease duration of patients were 10.1 ± 3.7 and 4.7 ± 0.8 years, respectively. The rate of missing values for PARQ/CARQ was generally satisfactory, test-retesting showed sufficient reliability. PARQ/CARQ mean child ability total scores (0–100, 100 = best) for medication were 73.1 ± 23.3/76.5 ± 24.2, for exercise: 85.6 ± 16.5/90.3 ± 15.0, for splints: 72.9 ± 24.2/82.9 ± 16.5. Construct validity was supported by PARQ and CARQ scores for medications, exercise and splints showing a fair to good correlation with the Global Adherence Assessment (GAA) and selected PedsQL scales. Adolescents showed poorer adherence than children. About one third of the parents and children reported medication errors. Perceived helpfulness was highest for medication, and adverse effects were reported the greatest barrier to treatment adherence.

**Conclusions:**

The German versions of the PARQ and CARQ appear to have a good reliability and sufficient construct validity. These questionnaires are valuable tools for measuring treatment adherence, identifying potential barriers and evaluating helpfulness of treatments in patients with JIA.

**Supplementary Information:**

The online version contains supplementary material available at 10.1186/s12969-023-00811-0.

## Background

Pursuant to the International League of Associations for Rheumatology (ILAR) criteria, the generic term *juvenile idiopathic arthritis* (JIA) comprises all types of inflammatory joint diseases of unknown cause in patients under 16 years of age that last at least 6 weeks [1]. In industrialized countries, JIA is the most common chronic inflammatory rheumatic disease in pediatric care. JIA often persists over a long period of time and in about half of the cases beyond adolescence [2–7]. Two recent outcome studies have shown that after about 17 years of disease only 40% and 33% of patients, respectively, are in drug-free remission [8, 9].

JIA patients are required to consistently follow complex treatment plans over the long term. A key element in managing this disease and influencing its outcome is adherence to prescribed treatments [10–15]. There are various methods to assess adherence, including electronic monitoring, drug assays, pharmacy refills, pill counts, and a variety of self-report measures. Adherence rates have been found to vary among these: self-report questionnaires have been shown to indicate higher adherence rates, suggesting an overestimate compared to more objective methods [13]. However, questionnaires seem to be the most feasible method for clinical routine although they are not fully reliable [16]. The Parent Adherence Report Questionnaire (PARQ) and the Child Adherence Report Questionnaire (CARQ) were developed to collect information on adherence in children with JIA and their parents and to determine risk factors for non-adherence and barriers to treatment. Both were evaluated as valid and reliable instruments, providing important information on benefits and problems with different treatment regimens [17–20].

The purpose of this work was to cross-culturally adapt and validate the PARQ and CARQ in German language and thereby gather information on adherence in a large German prospective multicenter inception JIA cohort of children and adolescents in the first years after diagnosis.

## Patients and methods

### Study design

The Inception COhort of Newly diagnosed patients with JIA (ICON), a, prospective observational cohort study, was used to evaluate the PARQ and CARQ. ICON aimed to observe patients with recent-onset of JIA according to the ILAR classification criteria [1] for at least 10 years. Eleven of the largest pediatric rheumatology centers in Germany recruited patients for this project from 2010 onwards and documented clinical characteristics and treatments by a standardized physician questionnaire every 3 months during the first year of observation and every 6 months in the subsequent years until the end of 2021. More details on the ICON cohort were described by Sengler et al. 2015 [21].

The PARQ and CARQ, global assessments of adherence (GAA) to drug treatment, exercises and wearing splints, and other questionnaires were completed by parents and patients (if age ≥ 8 years) 48 months after enrolment in ICON.

Between 2010 and 2012, 951 children and adolescents with JIA were enrolled in the ICON study. By the survey date (4-year follow-up) of PARQ and CARQ, there were *n* = 176 drop-outs (18.5%). Of the resulting 775 available patients, *n* = 243 did not participate in the 4-year follow-up but did participate in subsequent visits and thus remained under further observation. Thus, a total of 532 patients and their parents were available to complete PARQ and CARQ as part of ICON.

### Measures

Sociodemographic and clinical information such as age, sex, parents’ country of origin, disease onset, date of diagnosis, JIA category and global assessment of disease activity (measured by numerical rating scale, NRS 0–10) were provided by the treating pediatric rheumatologist. Patient-reported outcomes included the assessment of overall well-being and pain using NRS. Functional ability of the patients was assessed by the German version of the C-HAQ [22]. Health-related quality of life was reported along with the German versions of the Pediatric Quality of Life Inventory (PedsQL) generic core scales and rheumatology module scales (range 0–100, best = 100). The PedsQL rheumatology module assesses treatment-related problems with 7 single items [23].

Disease activity was measured by the clinical Juvenile Arthritis Disease Activity Score-10 (cJADAS-10), which includes the physician's global assessment of disease activity, the patient's global assessment of general well-being and the number of active joints (up to a maximum of 10). The 2014 JADAS-cutoff values for oligo- and polyarthritis were applied dependent on the cumulative joint involvement [24, 25].

The socioeconomic status of the patients was calculated using an established German multidimensional aggregated index [26]. As the parental work status is not assessed in the ICON study, the calculation of this index was modified to be based only on the parental educational level (including school education and vocational training) and the net household income. The study by Listing et al. includes more details [27]. The lower and upper quintiles of the sum of the education and income scores (6.55, 12.1) were used as cut-off points to define low, moderate, and high SES.

### Cross-cultural adaption and validation

The German versions of the PARQ and the CARQ were fully translated from its original Canadian- English version and cross-culturally adapted according to international guidelines [28, 29]. Two forward- and backward translations each were conducted by an expert committee consisting of a health scientist, three pediatric rheumatologists whose mother tongue was the target language (German) as well as an American-English native speaker with no medical background. Each translator performed a separate translation before a consensus version was implemented.

### Test–retest reliability

Families who completed the 4-year follow-up between December 2015 and December 2016 were sent the PARQ and CARQ (for children ≥ 8) again about 2 weeks later for retesting in complete.

### Adherence to treatment

The PARQ and CARQ were distributed to parents and patients, respectively, enrolled in ICON.

The CARQ, which is a simplified version of the PARQ, addressing children ≥ 9 years, has been pretested in both English and French [19, 20]. In our study, children ≥ 8 years were asked to fill out the CARQ as the ICON questionnaires are distributed age-group wise and the corresponding age group includes children from 8 years onwards.

The PARQ and CARQ are composed of three sections, each of which commences with a question including the phrase “in the past 3 months”, which is intended to emphasize the time since the last prescription of treatment.

Background section identifies who is primarily responsible for different treatment components: taking medication, doing exercises, and wearing splints.

Patients and methods section measures the child’s ability related to i) general level of difficulty in following treatment, ii) frequency of following treatment and iii) negative reactions in response to treatment which is evaluated with a 100 mm Visual Analogue Scales (VAS, 100 = “very hard” for item i and “always” for items ii) and iii). Responses to items i) and iii) are reversed so that higher scores indicate fewer difficulties and fewer negative reactions, respectively. An overall score for the child's abilities (Child Ability Total Score = CATS, 0—100) can be derived by averaging the answers to each of the three items. Furthermore, it is asked whether errors were made in medication behavior using a yes/no format: (1) ever forget to take medicine; (2) being careless at times about taking medicine; (3) when feeling better sometimes stopped taking medicine; and (4) when feeling worse when taking medicine, sometimes stopped taking it. Positive responses are summed, with total scores ranging from 0 (no errors) to 4.

Results section asks about caregiver´s and child`s perception regarding the helpfulness of each treatment component (e.g., medication, exercises, and splints) using a 100-mm horizontal VAS (100 = “very helpful”). Caregivers are also asked about the therapies they most preferred. The final question (only PARQ) inquires about potential barriers that families have encountered with regard to treatment.

Global Adherence Assessment (GAA) by parents and patients ≥ 13 years of age was assessed by asking how frequently i) medications, ii) exercises, and iii) splints were taken, performed, or worn, respectively. Responses were documented on a self-constructed 5-point Likert scale for each measure. Response options ranged from "almost always," "frequently," "sometimes," "rarely," to "not at all" and included "no therapy required."

### Statistical analyses

Standard descriptive statistics were used to describe the distribution of the single PARQ and CARQ items. The rate of missing values was calculated for each PARQ and CARQ item The basic population for the investigation of missing values was always the patients who had been prescribed the respective form of therapy.

Construct validity was investigated based on the PARQ and CARQ by assessing convergent and discriminant validity.

Within the assessment of convergent validity, scales measuring the same construct were correlated with each other: For each treatment component (medications, exercises, splints), the Spearman rank correlations were calculated for each of the VAS "frequency of adherence" and the CATS in relation to the GAA. In addition to that, we assessed polychoric correlations between the yes/no format question on errors in medication behavior: “when feeling worse when taking medicine, sometimes stopped taking it” and the PedsQL treatment subscale single item “medicine makes child feel sick”. For the assessment of exercises, we looked at Spearman rank correlations between the VAS iii) “negative reactions in response to treatment” and the PedsQL treatment subscale single item “physical therapy or daily exercises hurt”.

Within the assessment of discriminant validity, our underlying hypotheses were that some parameters are associated with better adherence: a younger age (since the parents can "control" more), a higher disease activity as well as more pain (since the burden of suffering is higher and thus also the expectation of an improvement of the state of health through e.g. medication), a higher SES (because therapies may also be carried out that cost the patients or their parents extra money), as well as a shorter duration of therapy with methotrexate (because with longer therapy the rate of patients with MTX aversion increases and thus presumably more patients no longer want to take MTX).

Different groups were formed based on clinical and sociodemographic parameters and correlated with the CATS for each corresponding treatment type. The groups included in our analyses were age ($\le 12\mathrm{\;years\;vs}. >12 \mathrm{\;years})$, sex (male vs. female), socioeconomic status (low, moderate, high), treatment with methotrexate (MTX, < 2 years vs. ≥ 2 years), pain (NRS = 0 vs. NRS > 0, overall well-being (NRS = 0 vs. NRS > 0), functional limitations (CHAQ = 0 vs. C-HAQ > 0) as well as disease activity (cJADAS ≤ 1 vs. cJADAS > 1). T-tests (Welch test) were performed to evaluate the ability of the PARQ and CARQ for their discrimination between selected patient groups with a hypothesized difference in adherence. To evaluate concordance of these questionnaires, comparisons of the CATS PARQ with the CATS CARQ were performed using a paired t-test.

Test–retest performance was carried out by the kappa coefficient and intraclass correlation coefficient (ICC) for categorical and continuously distributed variables, respectively. ICC values greater than 0.90 were considered excellent, between 0.75 and 0.90 good; whereas values between 0.50 and 0.75 or less than 0.50 were considered moderate and poor agreement, respectively [30]. As suggested by Landis and Koch [31] we regarded kappa coefficients below 0.21 as indicative of slight agreement, values from 0.21 to 0.40 as speaking for fair agreement, values between 0.41 and 0.60 as moderate, from 0.61 to 0.8 substantial and kappa values > 0.81 indicating almost perfect agreement.

## Results

### Patient characteristics

In total, after 4 years of observation, data for the PARQ and the CARQ were available for 481/532 parents and their children (*n* = 465), respectively. The demographic and clinical characteristics of the patients presented in Table 1 did not differ from those of the total ICON cohort (data not shown). For 78% of both parents, the country of origin was Germany, for 3% each Turkey and Russia. According to remaining data, 6 mothers and 6 fathers reported their countries of origin being Mongolia, Vietnam, Haiti, Sri Lanka, Philippines, Chile, India and Namibia.

**Table 1:** Sociodemographic and clinical characteristics of the study sample

Parameters	PARQ	CARQ
**N**	481	465
**Female, N (%)**	337 (70.1)	324 (69.7)
**Age at assessment, years**	10.1 ± 3.7	10.2 ± 3.7
**Age ≤ 12 ys, N (%)**	352 (73.2)	337 (72.5)
**Disease duration, years**	4.7 ± 0.8	4.7 ± 0.8
**JIA categories, N (%)**		
**Systemic arthritis**	14 (2.9)	12 (2.6)
**Oligoarthritis, extended**	71 (14.8)	69 (14.8)
**Oligoarthritis, persistent**	163 (33.9)	161 (34.6)
**RF- negative polyarthritis**	143 (29.7)	135 (29.0)
**RF- positive polyarthritis**	7 (1.5)	6 (1.3)
**Enthesitis-related arthritis**	34 (7.1)	34 (7.3)
**Psoriatic arthritis**	24 (5.0)	24 (5.2)
**Undifferentiated arthritis**	25 (5.2)	24 (5.2)
**Medication, N (%)**		
** NSAIDs**	94 (19.5)	89 (19.1)
** Glucocorticoids, systemic**	18 (3.7)	16 (3.4)
** DMARDs, total**	292 (60.7)	278 (59.8)
** Methotrexate**	223 (46.4)	213 (45.8)
** Other csDMARDs**	37 (7.7)	37 (8.0)
** Etanercept**	61 (12.7)	58 (12.5)
** Adalimumab**	48 (10.0)	48 (10.3)
** Other bDMARDs**	27 (5.6)	22 (4.7)
** Combination of bDMARD + MTX**	75 (15.6)	73 (15.7)
** No DMARD therapy (last 6 months)**	186 (38.7)	184 (39.6)
** Physician global assessment of disease activity (PhGA), NRS 0–10**	0.7 ± 1.3	0.7 ± 1.3
** PhGA = 0, N (%)**	272 (57.4)	266 (58.1)
** cJADAS-10 (range 0–30)**	2.6 ± 3.4	2.5 ± 3.2
** cJADAS-10 ≤ 1, N (%)**	237 (50.2)	229 (50.2)
** Patient global overall-wellbeing (PGA), NRS 0–10**	1.3 ± 1.8	1.3 ± 1.7
** PGA = 0, N (%)**	185 (38.6)	178 (38.4)
** Pain, NRS 0–10**	1.0 ± 1.8	1.0 ± 1.8
** Pain = 0, N (%)**	256 (53.4)	247 (53.4)
** Functional status, CHAQ (0–3)**	0.2 ± 0.4	0.2 ± 0.4
** CHAQ = 0, N (%)**	331 (69.4)	320 (69.4)
** Health related quality of life (PedsQL 4.0 total score, 0–100)**	87.4 ± 13.8	89.7 ± 12.2
**PedsQL 3.0 Rheumatology Module (Score 0–100)**		
** PedsQL pain and hurt**	86.4 ± 18.9	88.4 ± 18.5
** PedsQL daily activities**	96.9 ± 9.2	97.2 ± 8.4
** PedsQL treatment**	78.4 ± 21.9	81.2 ± 21.8
** PedsQL worry**	88.2 ± 19.0	91.3 ± 16.3
** PedsQL communication**	82.9 ± 24.3	85.2 ± 22.4
**Socioeconomic status**		
** - low, N (%)**	217 (45.1)	207 (44.5)
** - medium, N (%)**	156 (32.4)	152 (32.7)
** - high, N (%)**	108 (22.5)	106 (22.8)

Values are presented as mean ± standard deviation, unless otherwise stated

*PARQ* Parent adherence report questionnaire, *CARQ* Child adherence report questionnaire, *JIA* Juvenile idiopathic arthritis, *RF* Rheumatoid factor, *NSAIDs* Non steroidal anti-inflammatory drugs, *DMARD* Disease-modifying anti-rheumatic drugs, *cs* conventional synthetic, *b* biological, *PhGA* Physician’s global assessment of disease activity, *PGA* Patient’s global assessment of well-being, *cJADAS* Clinical Juvenile Arthritis Disease Acitivity Score, *NRS* Numeric rating scale, *CHAQ* Child Health Assessment Questionnaire, *PedsQL* Pediatric Quality of Life Inventory

Mean age at JIA onset was 5.4 years (SD 3.6) and mean age at enrolment into ICON was 6.1 years (SD 3.7). At assessment, a total of 357 patients were prescribed a medical measure: With 89%, the majority of patients received drug therapy (*n* = 316, including 46% methotrexate [MTX], 28% biologic disease modifying antirheumatic drugs [bDMARDs] and 16% combined treatment with bDMARDs and MTX). The cumulative duration of therapy was on average, 34.3 months (SD 17.3) for MTX and 13.4 months (SD 16.7) for a bDMARD. Almost 60% of patients were prescribed exercises/physiotherapy (*n* = 202), and 21% of patients were instructed to wear splints (*n* = 75).

The GAA by the parents and the children each revealed best values in drug therapy. Ninety-four percent of children (*n* = 63) reported having taken their medication “almost always” or “frequently”, whereas the results for exercises and splints were 79.5% (*n* = 31) and 83.4% (*n* = 15), respectively. Almost 8% of children (*n* = 3) reported that they “never” did their exercises and98.7% of parents (*n* = 282) stated their children “almost always” or “frequently” took their medication as prescribed, results for exercises were 90.4% (*n* = 159) and for splints 73.1% (*n* = 30).

### Missing values

The rate of missing values was acceptable for both PARQ and CARQ with a similar distribution throughout all sections concerning medication and exercises (values ranging from 1.9% to 15.8%, additional file, Table 1). Most of the missing values in both questionnaires were related to splint statements (41.3% on average for PARQ and 31.6% for CARQ). Fewest missing values (< 5%) were found for the 4 yes/no format questions assessing errors in medication behavior in both children and parents.

### Characteristics of the PARQ and CARQ

Regarding responsibility for each type of treatment, caregivers and patients reported that parents (mother, father, both) were primarily responsible for adherence to treatment recommendations. Table 2 shows the descriptive data of PARQ and CARQ.

**Table 2:** Characteristics of the PARQ and CARQ

	PARQ (*n* = 357)			CARQ (*n* = 185)		
**Questionnaire items**	Medication	Exercise	Orthopedic splint	Medication	Exercise	Orthopedic splint
Number of patients with the medical measure	316	202	75	167	98	43
**Part I: Responsibility for treatment, n (%)**						
Caregivers (mother, father, both caregivers)	271 (88)	161 (86.1)	33 (71.7)	97 (61.4)	54 (62.1)	11 (40.7)
Child only	23 (7.5)	17 (9.1)	10 (21.7)	29 (18.4)	21 (24.1)	12 (44.4)
Caregiver and Child	13 (4.2)	6 (3.2)	2 (4.4)	31 (19.6)	6 (6.9)	4 (14.8)
Others	1 (0.3)	3 (1.6)	1 (2.2)	1 (0.6)	6 (6.9)	0 (0.0)
**Part II: Child ability ( 0–100, 100 = best)**						
General level of difficulty in treatment, mean (SD)	70.8 (31.3)	83.1 (21.1)	66.0 (30.7)	73.9 (32.3)	89.0 (18.5)	88.2 (15.6)
Frequency following treatment, mean (SD)	89.7 (21.5)	88.7 (21.9)	76.0 (29.8)	86.2 (27.6)	89.7 (22.3)	74.0 (28.2)
Negative reactions in response to treatment, mean (SD)	59.3 (39.0)	84.8 (22.3)	78.7 (27.9)	72.3 (37.0)	93.8 (16.7)	86.7 (23.8)
Child ability total score (CATS), mean (SD)	73.1 (23.3)	85.6 (16.5)	72.9 (24.2)	76.5 (24.2)	90.3 (15.0)	82.9 (16.5)
Ever forgot to take medicine, n (%)						
Yes	79 (25.6)			33 (20.5)		
No	230 (74.4)			128 (79.5)		
Careless about taking medicine, n (%)						
Yes	36 (11.7)			21 (13.0)		
No	273 (88.4)			140 (87.0)		
Stopped taking medicine when feeling better, n (%)						
Yes	26 (8.4)			11 (6.9)		
No	284 (91.6)			149 (93.1)		
Stopped taking medicine when feeling worse, n (%)						
Yes	24 (7.8)			10 (6.1)		
No	283 (92.2)			153 (93.9)		
Any medication error (q1 to q4), n (%)	100 (32.3)			47 (28.7)		
Any medication error (q1 to q2), n (%)	82 (26.5)			38 (23.2)		
**Part III: Helpfulness of therapies** (0–100, 100 = best)						
Helpfulness of therapies, mean (SD)	87.4 (20.6)	84.8 (21.4)	80.8 (28.4)	83.6 (26.1)	86.9 (23.7)	73.5 (33.6)

*PARQ *Parent adherence report questionnaire, *CARQ *Child adherence report questionnaire, *CATS* Child ability total score

*SD* standard deviation, *q1 to q4* question 1 to question 4, *q1 to q2* question 1 to question 2

Overall, PARQ and CARQ scores were high both in parents and children.

Approximately one third of caregivers reported any error in medication behavior.

Helpfulness of therapies was perceived best for exercises from the perspective of children, whereas parents considered medication therapy the most helpful.

**Fig. 1:**
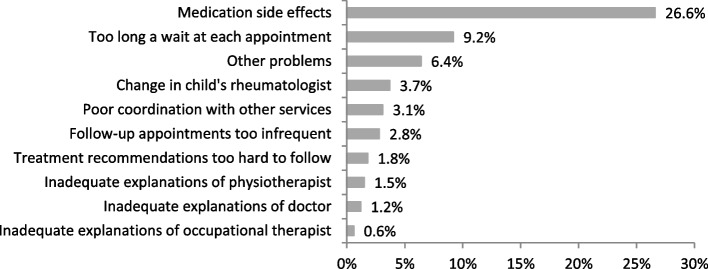
Parent-reported barriers to treatment adherence. Legend: Out of 357 parents of children with any medical measure (drug therapy, exercises or orthopaedic splints), 327 answered the questions regarding barriers in treatment adherence of their children. 128 (39%) reported barriers to their child's treatment adherence

About 40% of caregivers reported any barrier to treatment, with adverse effects from medications [26.6%, in patients with MTX (*n* = 96): 35.4%, in patients without MTX (*n* = 77): 7.8%] and too long waiting time at the doctor’s appointment (9.2%) being the most common (Fig. 1).

### Test–retest reliability

Questionnaires were readministered to 71 parents and 45 children after a mean of 13 ± 22 days (median 7 days, interquartile range 4 – 12 days) following the four-year follow-up. The response rate was 82%. Thus, test–retest reliability of the PARQ and CARQ was assessed in 58 parents and 39 JIA patients, respectively.

Intra-class correlation coefficients (ICC) for the medication scores showed moderate to excellent reproducibility (PARQ/CARQ, ICC = 0.69–0.96/ ICC = 0.53–0.75). ICC values for the exercise score ranged from poor to excellent concordance (PARQ/CARQ ICC = 0.28- 0.45/ ICC = 0–0.93). Splint scores’ reproducibility was also poor to excellent (PARQ/CARQ ICC = 0.01–0.90/ ICC = 0.00–0.93).

### Convergent validity

Correlations between the GAA with the VAS “frequency of following treatment” and the PARQ CATS for all three treatment domains were substantial to fair according to Landis & Koch [31] (medication: frequency: *r* = -0.37, CATS: *r* = -0.47; exercises: frequency: *r* = -0.25, CATS = -0.36; splints: frequency: *r* = -0.59, CATS: *r* = -0.68). Hence, the strongest correlation was found for splint therapy, indicating substantial agreement. Correlations between the PedsQL Treatment subscale and PARC CATS as well as CARQ CATS were slight to moderate.

When the answer to the yes/no format question on errors in medication behavior: “when feeling worse when taking medicine, sometimes stopped taking it” was correlated with the PedsQL 3.0 treatment subscale single item “medicine makes child feel sick”, a fair correlation was observed (*r* = -0.36).

Looking at exercises, correlations between the VAS “negative reactions in response to treatment” and the PedsQL 3.0 treatment subscale single item “physical therapy or daily exercises hurt” resulted in fair correlation (*r* = -0.29). For further results see Additional file, Table 2.

### Discriminant validity

Presumed differences in CATS-PARQ and CATS-CARQ for different patient groups could be detected but were not significant overall (see Additional file, Fig. 1).

When the questions whether errors were made in medication behavior using a yes/no format were evaluated in the PARQ and CARQ, significantly more medication errors were reported by parents of adolescents as well as adolescents themselves ≥ 13 years of age than parents of children and children themselves ≤ 12 years (see Table 3).

**Table 3:** Reporting of errors in medication by patients and parents according to age group of the patient

	**Parents of patients** ≥ 13 years	**Parents of patients** ≤ 12 years	**p**	**Patients** ≥ 13 years	**Patients** ≤ 12 years	
Q1: yes, n (%)	29 (38.7)	50 (21.4)	0.003	16 (23.9)	17 (18.1)	0.369
Q2: yes, n (%)	18 (24)	18 (7.7)	0.001	14 (21.2)	7 (7.4)	0.010
Q3: yes, n (%)	13 (17.3)	13 (5.5)	0.121	8 (11.9)	3 (3.2)	0.032

Q1: During the last 3 months, has your child ever forgotten to take his/her medication or have you forgotten to give your child his/her medication?

Q2: During the last 3 months, has your child been careless about taking their medication or have you been careless about giving your child their medication?

Q3: During the last 3 months, when your child got better, did he/she stop taking the medication or do you sometimes stop giving your child medication?

Further analyses showed that out of 87 patients who experienced adverse events (AE), 75 (86.2%) were treated with methotrexate (MTX). The average cumulative duration of MTX therapy in patients without AE (*n* = 240) was 33.5 months (SD 18.2), while patients who experienced AE (*n* = 87) were prescribed MTX for a mean of 39.6 months (SD 14.4) (*p* = 0.001). However, we did not see an association of adherence to MTX in PARQ-CATS or CARQ-CATS with duration of MTX therapy (MTX therapy < 2 years versus > 2 years (see Supplement Fig. 1).

### Concordance of PARQ and CARQ

**Fig. 2:**
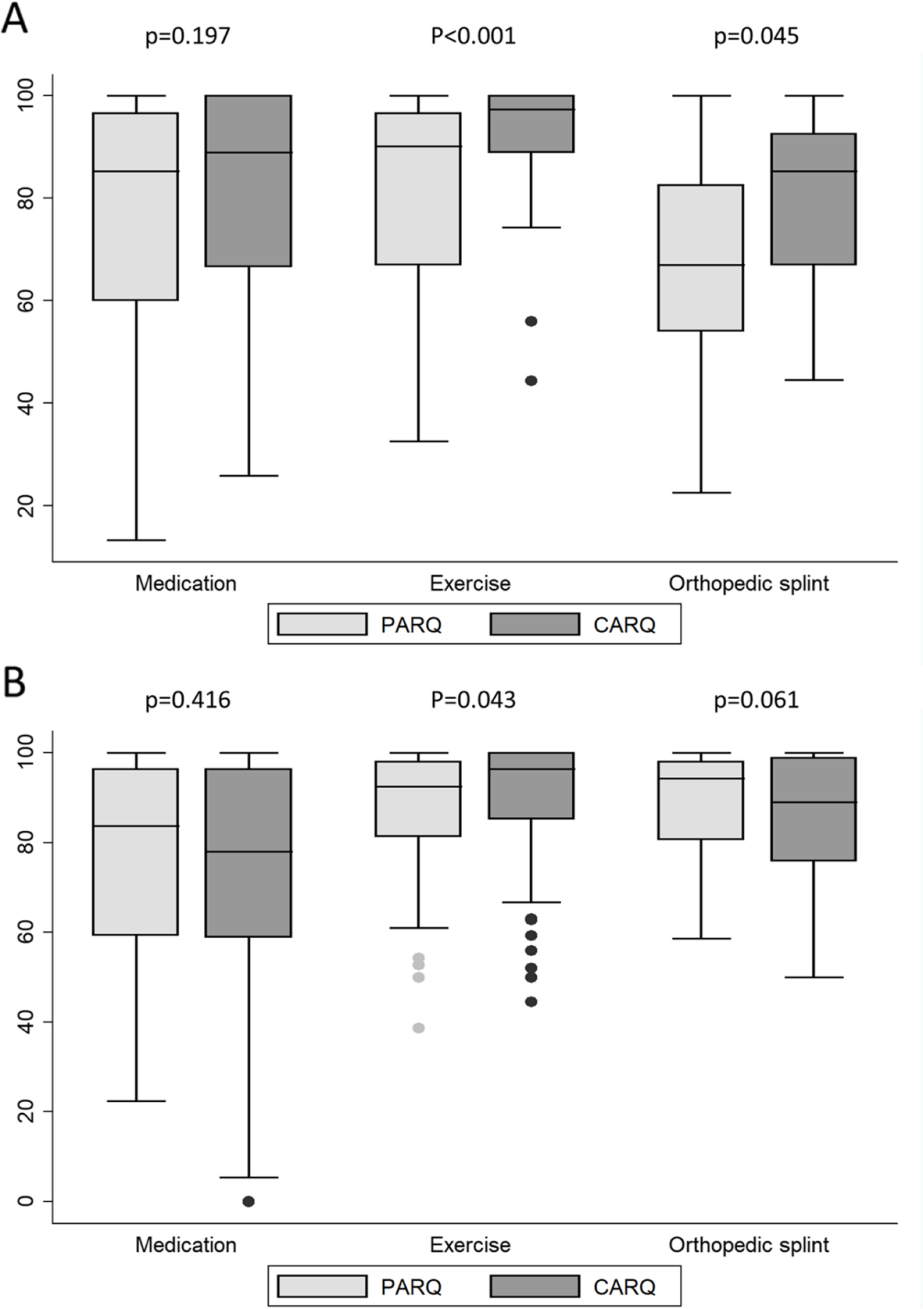
The Child Ability Total Scores (CATS) of PARQ and CARQ with respect to treatment types. Legend: Boxplots represent the distribution of the CATS values, with the horizontal line representing the median and the upper and lower ends of the box representing the 75th and 25th percentiles, respectively, for patients aged 8–12 years (**A**) and patients equal or above 13 years (**B**). For comparison of the PARQ and CARQ, patient proxy and self-reports were available of 63 pairs for medication treatment, 29 pairs for exercise treatment, and 13 pairs for splint therapy

In an additional analysis, the CATS of the PARQ was contrasted with the CATS of the CARQ with respect to the 3 treatment categories. The comparison of both measures showed good agreement regarding drug therapy but revealed significant differences for exercise therapy, which could be shown in patients 8–12 years (ΔPARQ—CARQ for the CATS was in mean -7.5 (SD 17.8), p50: -4.3, *p* < 0,001) and also in patients ≥ 13 years (Δ PARQ – CARQ for the CATS was in mean -11.6 (SD 16.2), p50: -5.0, *p* = 0.043). Results are presented in Fig. 2a and b.

## Discussion

This study determined the psychometric properties of the German versions of PARQ and CARQ in a large cohort of JIA patients. Both questionnaires were found to have satisfactory psychometric properties, making them reliable and valid tools for assessing treatment adherence, helpfulness of treatments, and detecting possible treatment barriers in patients with JIA.

Overall, PARQ and CARQ scores were high in our study with scores ranging mostly from 70 to 90, suggesting good overall adherence in this patient group. Our results are comparable to data presented by Lohse et al. [32] who found CARQ scores as well as PARQ scores in the same range (76.4 ± 21.2 and 74.8 ± 20.6, respectively) in children with JIA aged 8 to 16 years and their parents. PARQ and CARQ scores for medications, exercise and splints showed a fair to good correlation with the Global Adherence Assessment (GAA) and selected PedsQL scales thereby supporting construct validity.

Both the PARQ and the CARQ showed an acceptable rate of missing values apart from the questions regarding splint therapy, which counted most missing responses.

Whereas the intra-class correlation coefficients (ICC) for the medication scores showed moderate to excellent reproducibility, the ICC values for the exercise score and the splint score ranged from poor to excellent concordance. One interpretation of the results would be that the medication of patients with JIA remained mostly unchanged in the time between the two tests (test–retest), whereas the prescription of doing exercises or wearing splints was subject to greater fluctuation. In addition, the number of patients with the respective medical measures in the test–retest group is quite small, which leads to a large range when questions are answered differently in the two tests. Comparing the CATS of the PARQ with the CATS of the CARQ showed good agreement regarding drug therapy but significant differences for exercise therapy. One possible reason for this difference could be that the respective therapies are rated differently by children and their parents regarding helpfulness.

From the children's perspective, the usefulness of the therapies was rated highest for exercise, while parents found drug therapy most helpful, which is in line with data from April et al. [20]. Correspondingly, parents indicated adherence to medication best with PARQ score and GAA; consistent with the findings of Feldman et al. who reported a PARQ score for adherence to medication of 86.1 ± 26 in parents of Canadian JIA patients whereas the PARQ score for adherence to exercise (54.5 ± 31.6) was considerably lower than in our study [33].

Another treatment we studied was splint therapy, which is not widely used today, as reflected in the proportion of patients in our cohort who received this treatment (21%). Both parents and patients agreed that splint therapy was the least helpful treatment—an assessment that may also have influenced the incomplete responses to the relevant questions in the PARQ (many missings)—and the CATS score for splint therapy was the lowest from the parents' perspective.

For discriminant validity, several parameters were examined for their association with treatment adherence: The socioeconomic status (SES) as a major influence factor on adherence has been widely discussed in literature. Various studies suggest that a lower SES is associated with non-adherence [11–13]. A study by Rapoff et al. [34] describes the SES as the “most robust predictor of adherence”. The composition of the SES, however, differs from study to study, which must also be taken into account. Furthermore, healthcare systems may influence results: In Germany, health insurance is compulsory, and most prescriptions are covered by it. Our results did not show any influence of SES on adherence, as measured by the CATS, which agrees with the results of a Canadian study group [33].

There was no statistically significant difference in CARQ-CATS for any of the parameters examined (age,, sex, cJADAS, CHAQ, pain, general well-being), whereas in the PARQ lower general well-being and more pain led to higher adherence to exercise therapy as reported by parents.

Generally, JIA patients are fairly young at disease onset (5.4 years ± 3.6 in our study sample) which is why caregivers are highly and continuously involved into disease management from disease onset onwards. As such, younger patients were found to be more adherent [20]. Although we did not find significant differences regarding adherence to medication or exercise therapy comparing the CATS either of the PARQ or the CARQ in patients ≤ 12 years versus > 13 years, our analyses regarding medication intake (categorical questions, yes/no-type) indicated that older patients or adolescents who may no longer accept parental help reported significantly more errors than younger patients which coincides with other studies [13, 15, 20, 35]. However, we were unable to confirm the presumed association of adherence with duration of MTX therapy in our analysis. MTX is the most frequently used DMARD in JIA and is known to cause unfavorable side effects such as nausea [36] which is why patients are inclined to develop an aversion to it. The results from our study sample suggested a similar trend: more than 80% of patients with adverse events were treated with MTX and duration of MTX-therapy was longer for these patients compared to patients without experienced negative side effects. Also, according to parents, medication treatment caused most negative reactions and adverse events due to treatment was the most frequently stated barrier, which can be ascribed to MTX in any form of administration. Of the other more frequently named barriers, most were in the organisational area of the doctor's visit. In order to reduce these barriers, structural changes in outpatient clinical care would be necessary, which probably often fail due to the framework conditions.

There are a few limitations in this study: Using self-questionnaires remains a subjective method of measuring patient adherence, and we do not have objective measurement criteria for comparison available. As disease duration was similar among patients in ICON, we were unable to investigate the influence of this factor on treatment adherence. Our data show patient characteristics after 4 years of follow-up. Patients are expected to be well adjusted to their therapy by then and to have low disease activity or pain which is the case in our study sample. In addition, adherence appears to change, as may perceived barriers, so comparisons at different time points of prospective follow-ups would be desirable. As the questionnaire is quite long, it is more suitable for studies than for everyday clinical use.

## Conclusion

Both the German version of the PARQ and the CARQ appear to be valid and useful measures of adherence in JIA, providing important additional information on treatment adherence compared to general adherence measures, such as the GAA. The validation of the questionnaire in German language expands the circle of users, which may allow a more frequent evaluation of adherence and its relevance on the therapeutic success. Self-reported treatment adherence in JIA patients was good after 4 years of specialized care. The perceived usefulness of medications was rated highest, while adverse effects were reported as the greatest barrier to treatment adherence. These are important implications for good patient care, as possible side effects should be actively addressed and, if they occur, ways of alleviating them should be provided. Subsequent studies should focus on assessing, to what extent adherence influences the long-term outcome of JIA.

We will further investigate if and how adherence changes over time. With this comes the search for strategies to prevent non-adherence and further improve adherence in JIA patients so that the best possible outcome can be achieved.

## Supplementary Information

**Additional file 1: Table 1.** Distribution of missing values for PARQ and CARQ. **Table 2.** Convergent validity: correlations between CATS (VAS, PARQ, CARQ) and the GAA and PedsQL subscale items. **Figure 1.** Discriminant validity of PARQ (A) and CARQ (B) Child ability total score, displaying different groups with possible differences in adherence.

## Data Availability

The datasets analysed during the current study are not publicly available due to data protection reasons but are available from the corresponding author on reasonable request.

## Abbreviations

PARQ: Parent Adherence Report Questionnaire
CARQ: Child Adherence Report Questionnaire
JIA: Juvenile idiopathic arthritis
ICON: Inception Cohort Study of newly diagnosed JIA patients
GAA: Global Adherence Assessment
ILAR: International League of Associations for Rheumatology
NRS: Numerical rating scale
C-HAQ: Childhood Health Assessment Questionnaire
PedsQL: Pediatric Quality of Life Inventory
cJADAS-10: Clinical Juvenile Arthritis Disease Activity Score-10
VAS: Visual Analogue
CATS: Child Ability Total Score
ICC: Intraclass correlation coefficient
MTX: Methotrexate
