# Supplementary material for: Adherence, helpfulness and barriers to treatment in juvenile idiopathic arthritis – data from a German Inception cohort
Source: Pediatr Rheumatol Online J. 2023 Apr 12;21:31. doi: 10.1186/s12969-023-00811-0 (PMC10091650; doi:10.1186/s12969-023-00811-0)

Table 1: Distribution of missing values for PARQ and CARQ.

|  | | PARQ | | | | CARQ |  |  |  |
| --- | --- | --- | --- | --- | --- | --- | --- | --- | --- |
|  |  | Total | Medication | Exercise | Orthopedic splint | Total | Medication | Exercise | Orthopedic splint |
|  |  | N (%) | N (%) | N (%) | N (%) | N (%) | N (%) | N (%) | N (%) |
| number of patients with the medical measure | | 357 | 316 | 202 | 75 | 185 | 167 | 98 | 43 |
| Part I |  |  |  |  |  |  |  |  |  |
|  | responsibility for treatment |  | 8 (2.5) | 15 (7.4) | 29 (38.7) |  | 9 (5.4) | 11 (11.2) | 15 (34.9) |
| Part II | |  |  |  |  |  |  |  |  |
|  | general level of difficulty in treatment |  | 14 (4.4) | 25 (12.4) | 30 (40.0) |  | 9 (5.4) | 9 (9.2) | 15 (34.9) |
|  | frequency |  | 19 (6.0) | 22 (10.9) | 31 (41.3) |  | 14 (8.4) | 8 (8.2) | 11 (25.6) |
|  | negative reactions in response to treatment |  | 20 (6.3) | 23 (11.4) | 31 (41.3) |  | 12 (7.2) | 9 (9.2) | 12 (27.9) |
|  | ever forgot to take medicine |  | 7 (2.2) |  |  |  | 6 (3.6) |  |  |
|  | careless about taking medicine |  | 7 (2.2) |  |  |  | 6 (3.6) |  |  |
|  | stopped taking medicine when feeling better |  | 6 (1.9) |  |  |  | 7 (4.2) |  |  |
|  | stopped taking medicine when feeling worse |  | 9 (2.9) |  |  |  | 4 (2.4) |  |  |
| Part III | |  |  |  |  |  |  |  |  |
|  | helpfulness of therapies |  | 35 (11.1) | 32 (15.8) | 34 (45.3) |  | 7 (4.2) | 9 (9.2) | 15 (34.9) |
|  | experienced barriers to treatment | 30 (8.4) |  |  |  |  |  |  |  |

Table 2: Convergent validity: correlations between CATS (VAS, PARQ, CARQ) and the GAA and PedsQL subscale items

|  | PARQ/CARQ Child ability total score | | |
| --- | --- | --- | --- |
|  | Medication | Exercise | Orthopedic splint |
| Global assessment of adherence (Likert scale) | -0.47/-0.45 | -0.36/-0.62 | -0.68/-0.42 |
| PedsQL treatment | 0.45/0.35 | 0.15/0.08 | 0.25/0.31 |
| PedsQl treatment subscale item Medicine:”Medicine makes child feel sick.” | -0.36/-0.39 | - | - |
| PedsQl treatment subscale item Injections:”Child gets scared about having needle sticks/shots | -0.38/-0.33 | - | - |
| PedsQl treatment subscale item Physiotherapy:”Physical therapy or daily exercise hurts.” | - | -0.29/-0.23 | - |

Absolute value of correlation: < 0.21 slight agreement; 0.21-0.40 fair agreement; 0.41-0.60 moderate agreement; 0.61-0.80 substantial agreement; > 0.80 almost perfect agreement.

Figure 1: Discriminant validity of PARQ (A) and CARQ (B) Child ability total score, displaying different groups with possible differences in adherence.


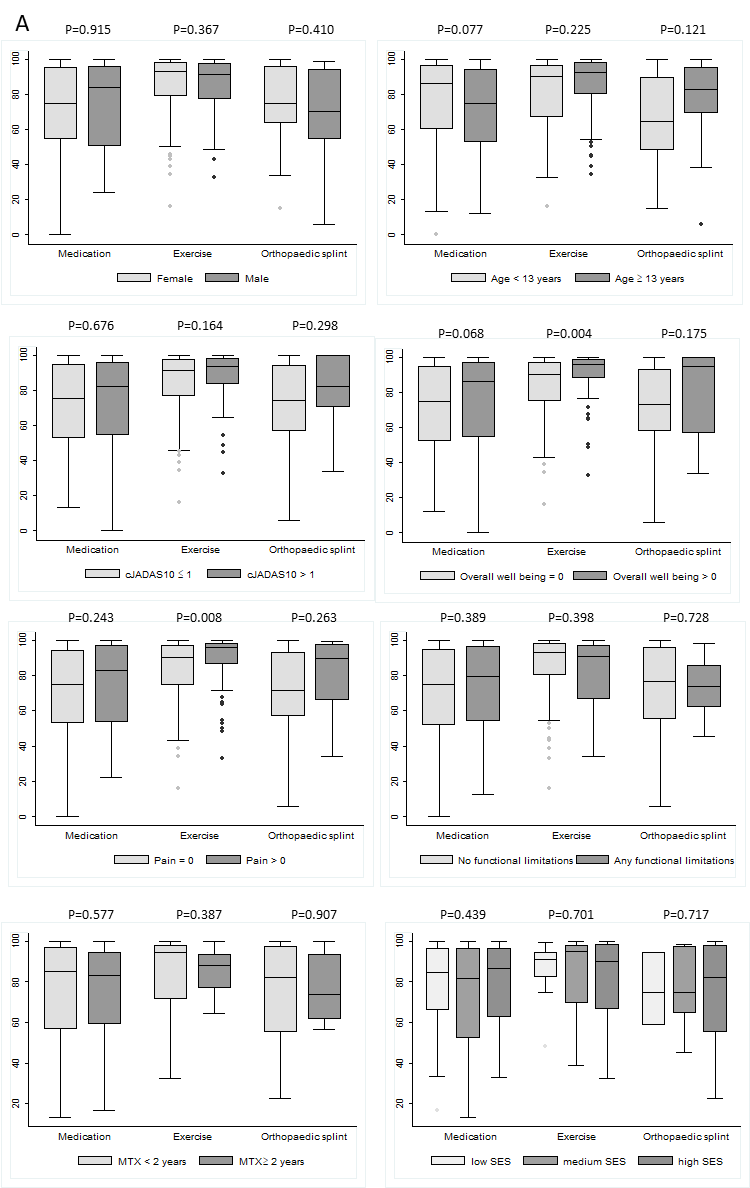

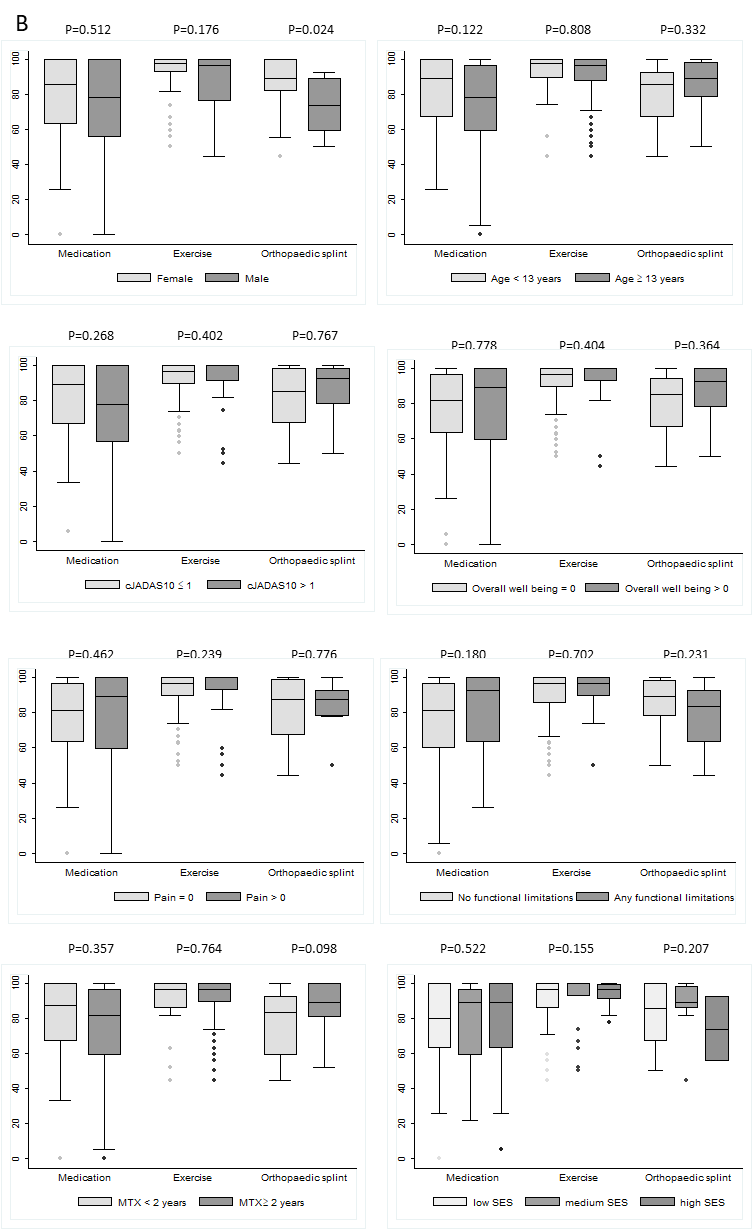

Supplement: Supplementary file 1 — Additional file 1: Table 1. Distribution of missing values for PARQ and CARQ. Table 2. Convergent validity: correlations between CATS (VAS, PARQ, CARQ) and the GAA and PedsQL subscale items. Figure 1. Discriminant validity of PARQ (A) and CARQ (B) Child ability total score, displaying different groups with possible differences in adherence. [file 12969_2023_811_MOESM1_ESM.docx]
